# Self-curling 3D oriented scaffolds from fish scales for skeletal muscle regeneration

**DOI:** 10.1186/s40824-022-00335-w

**Published:** 2022-12-22

**Authors:** Yong Shi, Xiaoxuan Zhang, Rui Liu, Xiaoyan Shao, Yuanjin Zhao, Zhuxiao Gu, Qing Jiang

**Affiliations:** 1 State Key Laboratory of Pharmaceutical Biotechnology, Division of Sports Medicine and Adult Reconstructive Surgery, Department of Orthopedic Surgery, Nanjing Drum Tower Hospital, The Affiliated Hospital of Nanjing University Medical School, 321 Zhongshan Road, Nanjing, 210008 Jiangsu People’s Republic of China; 2 Branch of National Clinical Research Center for Orthopedics, Sports Medicine and Rehabilitation, Nanjing, People’s Republic of China; 3 State Key Laboratory of Bioelectronics, School of Biological Science and Medical Engineering, Southeast University, Nanjing, 210096 China; 4 Department of Rheumatology and Immunology, Institute of Translational Medicine, The Affiliated Drum Tower Hospital of Nanjing University Medical School, Nanjing, 210002 China

**Keywords:** Bioinspired, Hydrogel, Cell orientation, Fish scale, Muscle regeneration

## Abstract

**Background:**

Volumetric muscle loss (VML) due to various reasons may cause motor dysfunction and tissue engineering has been proposed for muscle regeneration. However, developing three-dimensional (3D) tissue-engineered scaffolds that can mimic oriented cell growth of muscle tissues are challenging for regeneration medicine. Herein, we propose a novel self-curling 3D oriented scaffold (SCOS) composed of fish derived gelatin methacrylate (GelMA) and fish scales for repairing skeletal muscles.

**Methods:**

Fish scales of tilapia were decellularized and decalcified. Then, SCOSs were constructed by ultraviolet-coating methylated fish gelatin on the back of fish scales. C2C12 myoblasts were cultured on SCOSs, and after induction of myogenic differentiation, SCOS/C2C12 transplants were prepared for in vivo experiments.

**Results:**

Decellularized and decalcified fish scales (DDFSs) became soft and retained the original oriented microgroove surface structure that could induce oriented cell growth. SCOSs could self-curl into 3D structures when immersing in culture medium due to different swelling properties of fish GelMA and DDFSs. Cell experiments demonstrated that SCOSs enhanced the oriented growth and myogenic differentiation of C2C12 myoblasts. By integrating SCOSs and myogenic differentiated C2C12 myoblasts, the resultant SCOS/C2C12 transplants promoted de novo muscle regeneration and functional restoration of muscle activity in the mouse model of VML.

**Conclusions:**

Our results suggest that SCOSs loaded with myogenic differentiated C2C12 myoblasts can promote muscle regeneration in mice with skeletal muscle injuries, indicating application prospects of such scaffolds in muscle tissue engineering and other related fields.

**Supplementary Information:**

The online version contains supplementary material available at 10.1186/s40824-022-00335-w.

## Background

Skeletal muscle is an important body tissue responsible for movement, postural support, thermogenesis, and a series of metabolic functions [1]. Volumetric muscle loss (VML) due to traumatic injury, infection, or tumor ablation is one of the leading causes of motor dysfunction, which is an urgent problem in the field of muscle function rehabilitation [2–4]. Although the transplantation of autologous muscle flaps is currently a treatment approach, it has risk of donor site morbidity and may fail to effectively reconstruct functional skeletal muscle tissue [3, 5]. To solve these problems and further promote VML treatment, various tissue engineering materials such as bio-derived materials like muscle extracellular matrix nanofibrils and synthetic materials like polycaprolactone have been developed, and significant progresses have been made [6, 7]. However, these biomaterials may have immunological rejection because of the application of heterologous tissues or the material preparation technology [8]. Besides, due to the directional nature of muscle growth, many engineered tissues can hardly simulate the native three-dimensional (3D) microstructures or construct aligned muscle fibers. Therefore, the development of novel biomaterials with good biocompatibility, low immunogenicity, and specific 3D structures and functions is still urgent in the field of VML treatment.

**Fig. 1:**
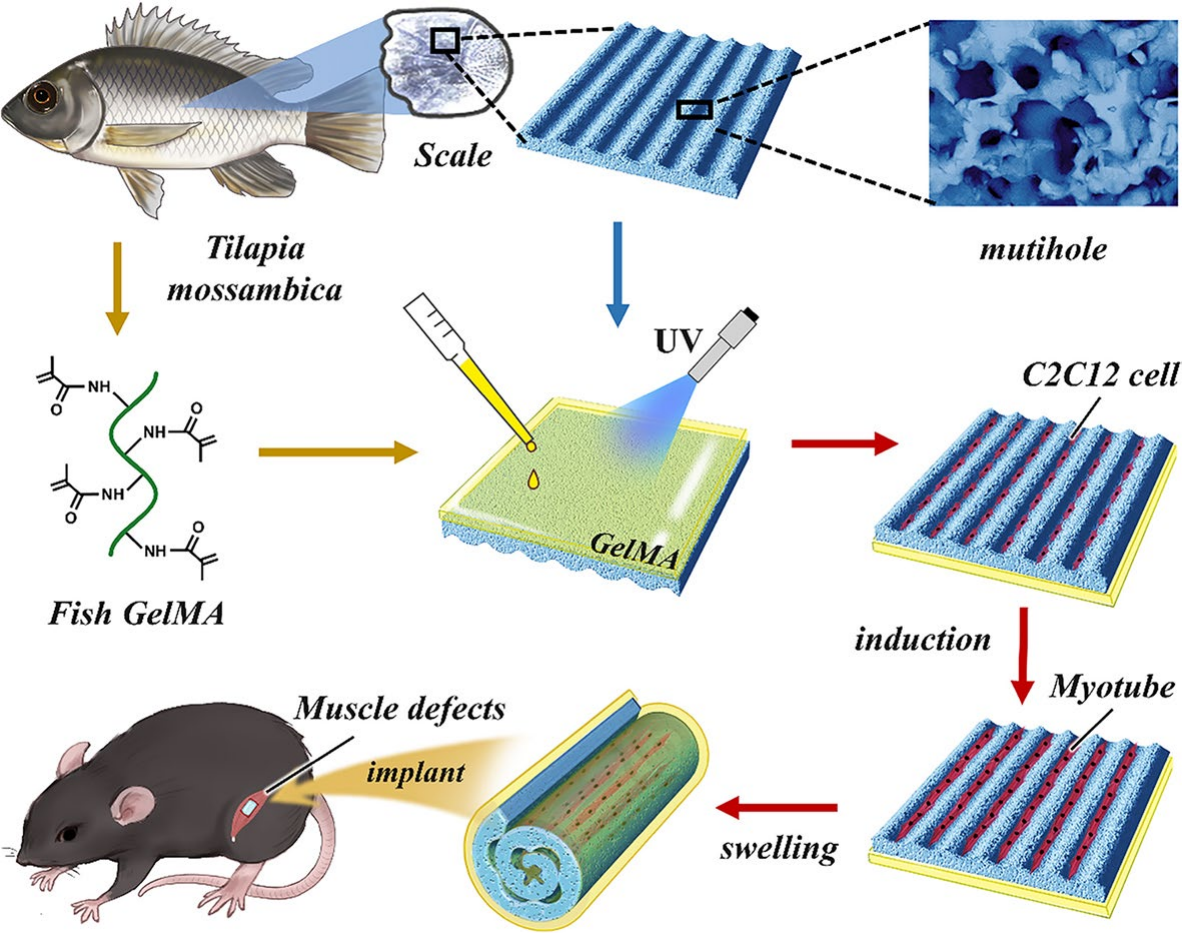
Schematic illustrations of the SCOS/MT fabrication for VML treatment

In this study, inspired by the scales of tilapia, we propose a novel self-curling hybrid scaffold from fish scales with desired features for promoting skeletal muscle regeneration, as schemed in Fig. 1. Due to the great biocompatibility and convenient accessibility of fish-derived materials, fish scales and fish skin gelatin have been widely used in bioengineering materials [9–11]. Specially, the specific microstructure on the surface of fish scales can induce oriented cell growth, which is of great significance for tissue engineering [12]. In addition, it has been reported that fish scale-derived collagen matrix is not immunogenic and allows excellent cell growth, and thus may serve as a scaffold to reconstitute tissues including cornea and skin [10, 12]. Also, by grafting methacrylate onto the fish gelatin, the obtained fish GelMA could form soft hydrogels under ultraviolet [13]. Despite the recent achievements of these fish-derived biomaterials in many clinical areas, their applications in the field of skeletal muscle repair has seldom been reported. Besides, the current application is still limited to the planar structures of fish scales, and there are few reports on the application of fish scales in 3D tissue engineering scaffolds.

Herein, by integrating heterogeneous fish GelMA and fish scales, we develop a self-curling 3D oriented scaffold (SCOS) with low immunogenicity and cell orientation ability for the repair of skeletal muscle injury. To make the hydroxyapatite-rich and inflexible fish scales suitable for muscle engineering, they were firstly decellularized and decalcified [14]. With these processes, the natural stiff fish scales became softer, lighter, and almost transparent [15]. Notably, the decellularized and decalcified fish scales (DDFSs) were proved to mainly contain type I collagen, and retained the original oriented microgroove surface structure, inducing and facilitating oriented cell growth [16]. However, DDFS can only induce planar oriented growth. Considering the 3D oriented growth of muscle tissues, we need to construct a 3D scaffold with DDFS. By ultraviolet-coating the fish GelMA with different swelling properties on the back of DDFSs, the obtained fish GelMA hydrogel and fish scale hybrid scaffolds could self-curl into 3D structures when immersing in culture medium. Cell experiments revealed that the DDFS had great biocompatibility, and could enhance myogenic differentiation of C2C12 myoblasts as well as guide them to align into ordered myotubes. It was demonstrated that the SCOSs loaded with induced myotubes (SCOS/MT) could successfully induce de novo formation of skeletal muscle tissues after being transplanted into a murine model of VML. Based on all these features, it is believed that such SCOSs supply a novel idea for the research of skeletal muscle regeneration and find applications in other fields.

## Methods

### Decellularization and decalcification of fish scales

The fish scales harvested from tilapia were cleaned with distilled water for several times and decellularized according to the method described previously. Fish scales were first treated with 10 mM Tris-HCl buffer (Sigma) and 0.1% EDTA (Sigma) for 24 hours, and then they were left in Tris-HCl buffer containing 0.1% SDS (Sigma) at 4 °C for 3 days to eliminate the cellular components. The decalcification process was performed by immersing fish scales in 10% formic acid (Greagent) for 48 hours at room temperature. The decellularized and decalcified fish scales (DDFS) were finally sterilized and then stored in sterile PBS for further experiments.

### Mechanical strength measurement

The mechanical strength was determined with an Instron 2752–005 tensile-testing machine equipped with a load capacity of 100 N. Fish scales were cut into 3.0 mm × 6.0 mm and the assay was carried out at a rate of 10 mm/min at room temperature (*n* = 3). Then the values of Young’s modulus were calculated according to the stress and strain data.

### DNA and collagen content detection

Total DNA from fish scales with the same weight was extracted using a Universal Genomic DNA Purification Mini Spin Kit from Beyotime (D0063). And then the content of DNA extraction was detected. The collagen content of fish scales with the same weight was detected using a fish collagen (COL) ELISA kit from AiFang biological (AF69275-A).

### Fish GelMA synthesis and GelMA hydrogels preparation

To synthesize fish GelMA, the method as previously described was employed. First of all, fish gelatin was dissolved in DPBS (Sigma) by the 10% (w/v) ratio under constant stirring at 50 °C for 3 hours. Then the fully dissolved gelatin solution was added with 6% (v/v) of methacrylic anhydride (MA; Sigma-Aldrich, USA) in a dropwise manner under stirring conditions at 50 °C. After 3 hours of reaction, unreacted MA was removed by dialyzing for 1 week at 40 °C in distilled deionized water. The solution was subsequently lyophilized and the obtained GelMA was preserved at − 20 °C for further application. Different concentrations of GelMA solution were mixed with 1% (v/v) of 2-hydroxy-2-methylpropiophenone (HMPP; Sigma-Aldrich, USA) and then ultraviolet crosslinked for 2 minutes.

### Scanning electron microscopy and energy dispersive spectrometer

Gold-palladium were sputter-coated on the lyophilized fish scales, fish GelMA hydrogels and SCOSs, and the surface or cross-sectional morphologies were observed by scanning electronic microscope (SEM; Hitachi, TM-1000). To identify the morphology of myotubes loading on the DDFSs, they were fixed with paraformaldehyde and dehydrated with different concentrations of ethanol for subsequent processing. The surface elements of fish scales before and after decalcification were determined with the energy dispersive spectrometer (EDS).

### Mass swelling ratio examination

Mass swelling ratios of hydrogels with different concentrations of GelMA were evaluated using 4 cylindrical specimens. The specimens were immersed in PBS for 24 hours. After removing the excessive liquid gently, the swollen weight was weighed with an analytical balance. All specimens were then lyophilized for 48 hours to measure the dry weight. The swollen and dry weight of fish scales was also measured with the same method. The swelling ratio was calculated according to the equation below:

$$\textrm{Mass}\ \textrm{swelling}\ \textrm{ratio}=\frac{\textrm{Swollen}\ \textrm{weight}\ \textrm{of}\ \textrm{the}\ \textrm{sample}}{\textrm{Dry}\ \textrm{weight}\ \textrm{of}\ \textrm{the}\ \textrm{sample}.}$$

### Angle measurement of SCOS

The shape change degrees of SCOS after self-curling were measured using Image J software according to the method previously reported [17].

### Fourier-transformed infrared (FTIR)

FTIR spectra of freeze-dried fish GelMA, DDFS and SCOS were recorded using a Nicolet IS5 FTIR Spectrometer (Thermo). The spectra were obtained at different wavelengths from 4000 to 400 cm^− 1^ with 4 cm^− 1^ resolution.

### Cell culture

C2C12 mouse myoblasts (ATCC) cultured with DMEM containing 10% FBS and 1% penicillin/streptomycin were kept in the conditions of 37 °C and 5% CO_2_. To induce C2C12 myogenic differentiation, the culture medium consisting of DMEM, 2% horse serum and 1% penicillin/streptomycin was employed. After further culture for 5 days, the myotubes were successfully induced. All culture medium, FBS, and penicillin/streptomycin were from WISENT, and horse serum was from Gibco.

### Viability analysis of cells cultured on DDFS and SCOS

The viability of C2C12 myoblasts was evaluated with a Calcein-AM/propidium iodide (PI) Live-Dead Cell Staining Kit (CA1630, Solarbio life science, China). According to the supplied protocol, cultured cells were incubated with staining solution for 15 minutes away from light. Then, a fluorescence microscope (Nikon) was employed to take fluorescence photographs, and image analysis was conducted with ImageJ software. To detect cell proliferation ability, culture medium was added with 10% CCK-8 Cell Counting Kit solution (A311-02, Vazyme, China). After an incubation of 40 minutes, the absorbance at 450 nm was measured for analysis.

### Subcutaneous implantation of DDFS and SCOS

C57BL/6 mice were purchased from Ziyuan Biotechnology (Hangzhou, China), and all mice were kept in an animal care facility with temperature and 12 hours light/dark cycle controlled. Food and water were supplied following animal protection regulations. DDFSs or SCOSs were implanted subcutaneously for 2 weeks. Then mice were sacrificed to obtain serum and surrounding skin tissues along with DDFSs or SCOSs. Serum was harvested for biochemical analysis. Surrounding skin tissues along with DDFSs or SCOSs were fixed using 4% paraformaldehyde (Servicebio) and made into paraffin sections for hematoxylin & eosin (H&E), Masson trichrome and tumor necrosis factor α (TNF-α) immunohistochemical staining.

### Degradability test of DDFS and SCOS

Decellularized fish scales with or without decalcification were immersed in simulated body fluid (SBF) at 37 °C, and all samples were weighed every 2 days to detect the degradability. The percentage of weight loss was used to measure the degradation rate. SCOS was implanted under the skin of mice, and the implant along with the skin tissue was taken out to observe the remaining scaffold.

### Transplantation of the constructs in a VML mouse model

To generate the VML mouse model, 2-month-old C57BL/6 mice were anesthetized with an inhalation anesthesia machine using isoflurane, and then about 75% of the quadriceps femoris muscles were resected using a surgical blade, as reported previously. The DDFS/MT constructs with a 3 mm (x) × 6 mm (y) size, SCOS or SCOS/MT constructs with a 3 mm (x) × 6 mm (y) × 3 mm (z) size were implanted on the defect region, and the size of transplants was similar to the study previously reported [6]. VML mice without treatment were set as control. 8 weeks after transplantation, all mice were sacrificed for subsequent experiments.

### In vivo survival test of C2C12

To evaluate the survival condition of C2C12 myoblasts in vivo, VML mice were implanted with red fluorescent protein (RFP)-C2C12-loading SCOSs. The fluorescence signal was detected 7 days after implantation.

### Immunofluorescence and Immunohistochemical staining

For immunofluorescence staining, cultured cells and tissue samples were both fixed using 4% paraformaldehyde. Then, tissue samples were embedded in paraffin to prepare tissue slices (5 μm thickness). Paraffin sections were then rehydrated and treated with antigen retrieval solution (Sigma) at a sub-boiling temperature for 15 minutes followed by cooling down. After the process of permeabilization and blocking, all samples were incubated with primary antibodies (1:200), secondary antibodies (1:200) and DAPI (Beyotime). After several washings, immunofluorescence images were then photographed with a confocal microscopy (Olympus FV3000). To observe the cellular morphologies, F-actin of C2C12 cells was stained with FITC-phalloidin (Thermo) and DAPI.

For immunohistochemical staining, tissue sections were rehydrated and then treated with 3% H_2_O_2_ and antigen retrieval solution. After incubation with primary and second antibodies, the positive area was visualized with DAB HRP substrate. Sections were then counterstained with hematoxylin for cell nuclei. Primary antibodies were myogenin (MYOG; Abcam), myosin heavy chain (MyHC; Abclonal), α-smooth muscle actin (α-SMA; CST), acetylcholine receptor (AchR; Proteintech), and TNF-α (Abclonal). All secondary antibodies were from Proteintech.

### Western blot analysis

The whole protein extraction kit (KGP2100, KeyGEN, China) was used to extract protein lysates of C2C12 cells. After being separated by electrophoresis on 10% SDS–PAGE at 80 V for 40 minutes and 120 V for 60 minutes, protein samples were transferred to PVDF membranes (Millipore), followed by the process of blocking. PVDF membranes were then incubated with diluted primary (1:1000) and secondary (1:10000) antibodies, respectively. Finally, after several washings, protein expression was determined with the NcmECL High kit (NCM Biotech) and imaged by a Tanon 5200 Multi Scanning System (Tanon). Primary antibodies were myosin heavy chain (MyHC; Abclonal), myogenin (MYOG; Abcam), myoblast determination protein 1 (MyoD1; Abcam), and α-Tublin (CST). All secondary antibodies were from AiFang biological (AFSA001, AFSA004).

### Real-time quantitative polymerase chain reaction (RT-qPCR)

The UNlQ-10 Column Trizol Total RNA Isolation Kit (B511361, Sangon Biotech, Shanghai, China) was used to extract total RNA. The RNA quality and concentrations were then assessed, and the first-strand cDNA synthesis kit (Novoprotein, Shanghai, China; E041) was employed to synthesize cDNA. The ABI ViiA 7 Real-time quantitative PCR system (Thermo) was employed to perform Real-time Quantitative PCR with ChamQ SYBR Color qPCR Master Mix (Q411-02, Vazyme, China). The sequences of primer were exhibted in Table S1.

### Histological analysis

The process of prepare tissue slices were performed as previously mentioned. Then tissue slices were used for the H&E, Masson trichrome, and immunostaining. Images were taken by an Olympus IX71 microscope. Histological quantification was conducted with ImageJ.

### Gait analysis

Footprint patterns of mice were visualized by painting the hindfeet with non-toxic washable paint and placing the mice at the entrance of a dark tunnel (40 cm long 8 cm wide 8 cm high) over white paper. Gait analysis was performed 2 days 8 weeks after the surgery.

### Statistical analysis

All quantitative data presented was mean ± standard deviation (SD). Data analysis was conducted with GraphPad Prism 9. Two-tailed Student’s t-test and one-way ANOVA were adopted, and *p* value < 0.05 indicated significant difference.

## Results

### Fabrication of the self-curling 3D oriented scaffold

**Fig. 2:**
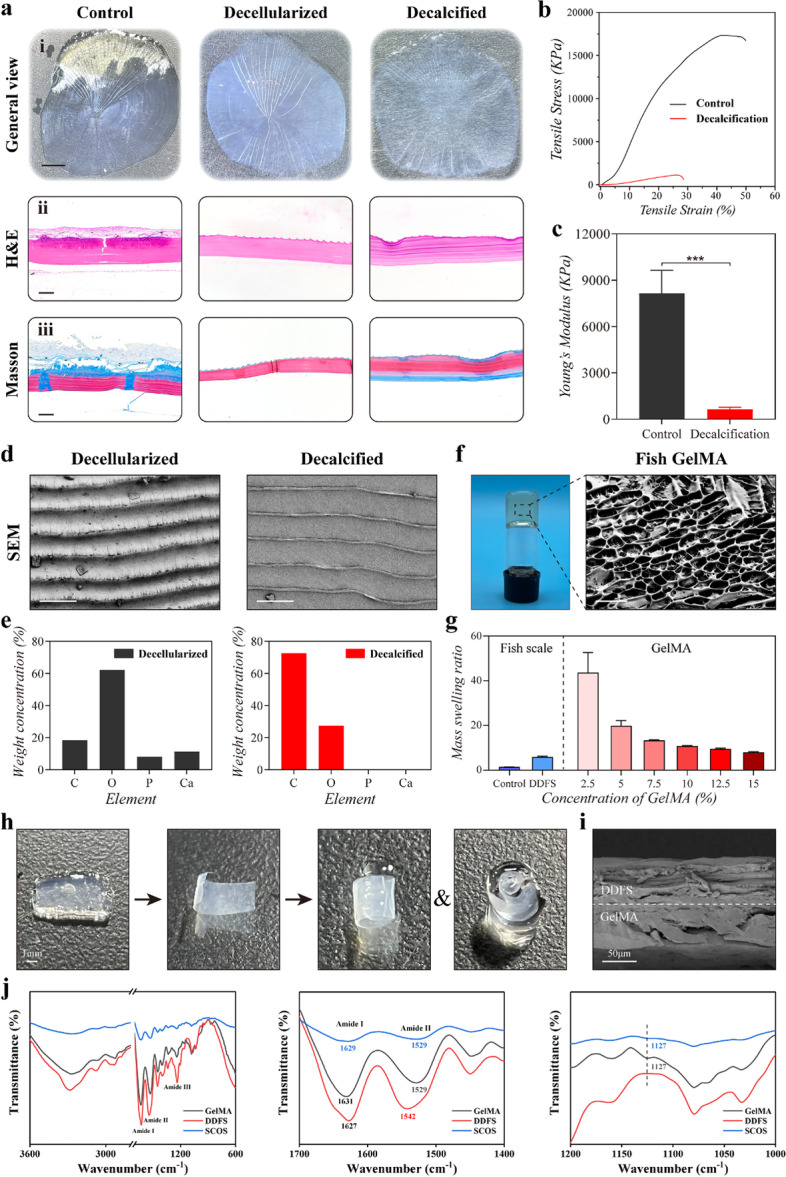
Characterization of DDFS and fish GelMA hydrogel. **a** (i) General observation of the fish scale before and after decellularization and decalcification (scale bar: 1 mm). (ii-iii) H&E and Masson trichrome staining of fish scales before and after decellularization and decalcification (scale bar: 100 μm). **b** The stress-strain curves of fish scales before and after decalcification. **c** The quantitative analysis of Young’s modulus (*n* = 3, mean ± SD). **d** SEM images of fish scales after decellularization and decalcification (scale bar: 100 μm). **e** EDS measurements of fish scales after decellularization and decalcification. **f** General observation and SEM image of hydrogels derived from fish GelMA (scale bar: 100 μm). **g** The mass swelling ratios of fish scales and hydrogels with different concentration of fish GelMA (*n* = 4, mean ± SD). **h** The self-curling process of DDFS and fish GelMA hydrogel hybrid scaffold (scale bar: 1 mm). **i** Cross-sectional SEM image of SCOS (scale bar: 50 μm). **j** FTIR analysis of fish GelMA hydrogel, DDFS and SCOS. ** *p* < 0.01, by two-tailed Student’s t test

In a typical experiment, fish scales from tilapia were first decellularized with Tris-HCl buffer containing 0.1% SDS for 3 days [18, 19]. After decellularization, fish scales were decalcified with formic acid for 3 days, and then DDFSs were obtained. The general view and histological analysis of fish scales were performed before and after decellularization and decalcification (Fig. 2a). H&E staining revealed that cells on fish scales were removed successfully (Fig. 2a). The DNA content of fish scales was almost undetectable after decellularization (Fig. S1a). DAPI staining of fish scales also confirmed that the cell component was removed after decellularization (Fig. S1c). The collagen components were shown by Masson trichrome staining and represented as blue (Fig. 2a). The collagen content of fish scales before and after decellularization and decalcification were also detected (Fig. S1b). Mechanical testing of fish DDFSs was then carried out (Fig. 2b). The Young’s modulus of fish scales decreased significantly after decalcification (Fig. 2c). The microstructures of DDFSs were observed by scanning electron microscope (SEM). We confirmed that calcium (Ca) and phosphorus (P) elements of fish scales were undetectable after decalcification (Fig. 2d, e and S1d) according to the energy dispersive spectrometer (EDS) measurements. The above results indicated that cellular components and hydroxyapatite were removed while the microstructures on DDFSs were retained. Then we used a scalpel to cut out the required part with oriented microstructures on the surface of the DDFSs for following experiments.

For fish GelMA preparation, methacrylic anhydride reacted with fish gelatin through dissolving them in DPBS at 50 °C according to the well-established synthesis process [13]. Representative ^1^H NMR spectrum was shown in Fig. S2. It could be found that the peak of amino lysine significantly decreased in fish GelMA after the synthesis process. The swelling behavior of fish GelMA hydrogels and DDFSs was then analyzed (Fig. 2f, g). Due to different swelling properties of DDFSs and fish GelMA hydrogels, the DDFS and GelMA hybrid scaffolds could self-curl and form a 3D structure after being immersed in the medium for a period of time, as shown in Fig. 2h. To determine the the appropriate concentration of fish GelMA, we measured the shape change degrees of SCOSs with different concentrations of fish GelMA after self-curling according to the method previously reported [17]. SCOSs with 5% fish GelMA were found to exhibit greater shape change compared to SCOSs with other concentrations of fish GelMA (Fig. S3a, b). Thus, 5% fish GelMA were finally chosen to light-cured on the back of DDFSs to prepare the scaffolds for further experiments. The mechanical test of SCOSs were then conducted (Fig. S3c). Cross-sectional SEM image showed the structure of SCOS composed of fish GelMA and DDFS (Fig. 2i). Fourier-transform infrared (FTIR) were used to confirm that DDFSs was coated with fish GelMA in SCOSs. Specifically, the FTIR spectra of fish GelMA, DDFS and SCOS all showed the characteristic peaks of amide I, II and III. SCOS showed the same characteristic peaks as fish GelMA at 1529 cm^− 1^ and 1127 cm^− 1^, which proved the successful coating of fish GelMA onto DDFSs (Fig. 2j).

### Biocompatibility and degradability test of DDFS and SCOS

**Fig. 3:**
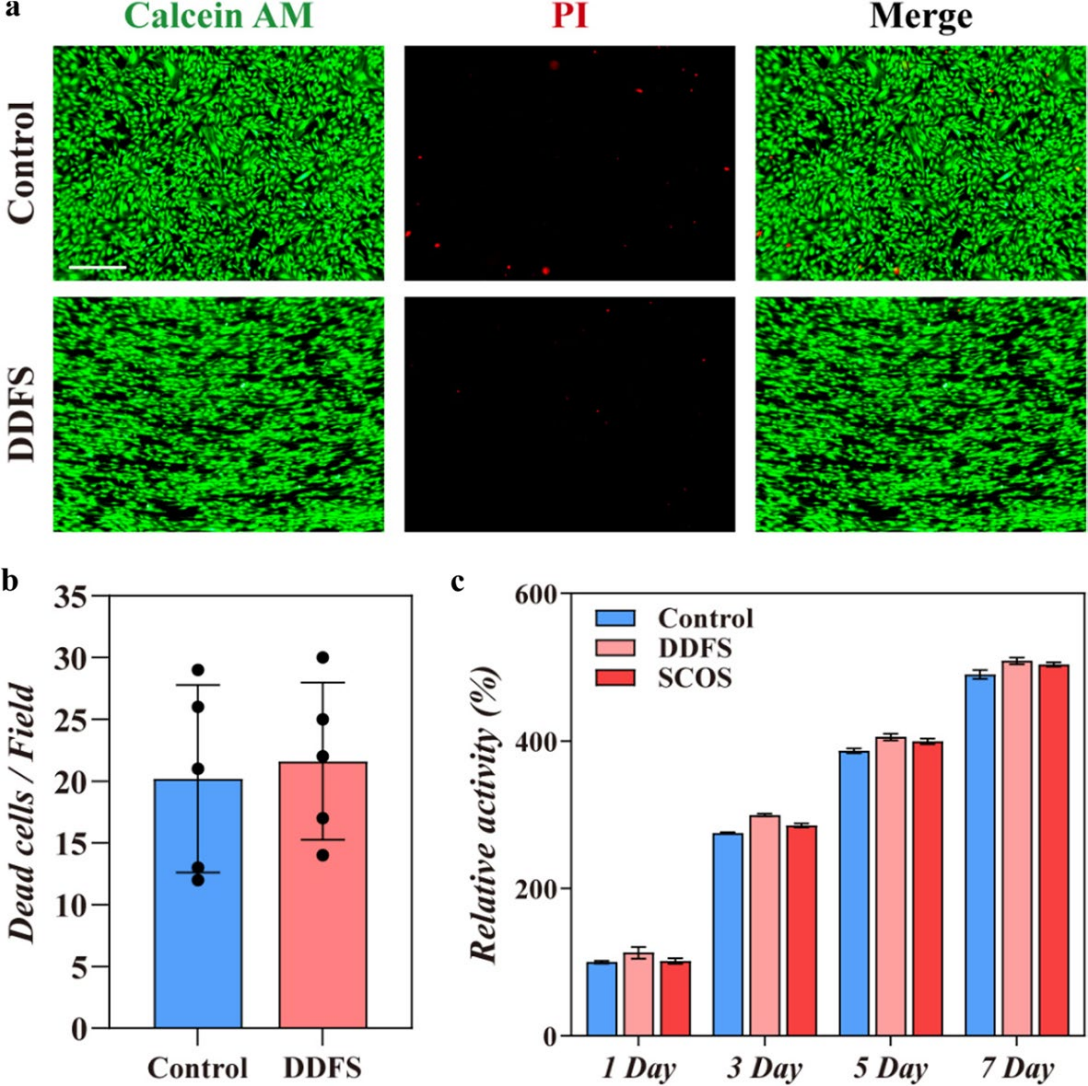
The in vitro biocompatibility of DDFS and SCOS. **a** Live/Dead cellular staining of C2C12 cultured on the cell culture plate (control) or DDFS (scale bar: 500 μm). **b** Quantitative results of dead cells (*n* = 5, mean ± SD). **c** Relative activity of C2C12 cultured on the cell culture plate (control), DDFS or SCOS by CCK-8 assay (*n* = 4, mean ± SD)

In vitro biocompatibility of DDFSs was determined by CCK-8 assay and Live/Dead cellular staining. The C2C12 myoblasts were seeded and cultured on the sterilized DDFSs. Live/Dead analysis indicated that C2C12 cells grew well with a normal and healthy morphology on DDFSs 24 hours after seeding (Fig. 3a, b). Then we performed the CCK-8 analysis to evaluate the viability of C2C12 myoblasts grown on DDFSs and SCOSs after 1, 3, 5 and 7 days. As shown in Fig. 3c, C2C12 cells growing on DDFSs and SCOSs exhibited similar viability to cells growing in blank wells. Furthermore, Live/Dead cellular staining revealed that differentiated C2C12 cells on DDFSs still maintained good cell viability (Fig. S4a, b). To evaluate the in vivo biocompatibility, DDFSs and SCOSs were subcutaneously implanted under the skin of mice. The skin tissue with implanted DDFSs and blood serum were harvested and prepared for histological analysis after 2 weeks. H&E and Masson trichrome staining indicated no inflammatory or necrotic response around the implanted DDFSs and SCOSs (Fig. S5a,b). Immunostaining for TNF-α showed that immune response caused by DDFSs and SCOSs was relatively mild (Fig. S5c). Biochemical detection of serum also revealed no significant differences in mice implanted with DDFSs or SCOSs (Fig. S5d). We then examined the degradability of DDFS by immersing DDFS in SBF, and found that DDFS had great degradation properties after decalcification (Fig. S1e). Furthermore, the in vivo degradability of SCOS was also detected according to the study previously reported [10]. It was difficult to observe SCOSs after being subcutaneously implanted under the skin of mice for 4 weeks, revealing good degradability of SCOS (Fig. S6). The above analysis showed that SCOS was suitable for tissue engineering.

### Oriented growth of C2C12 myoblast induced by DDFSs

**Fig. 4:**
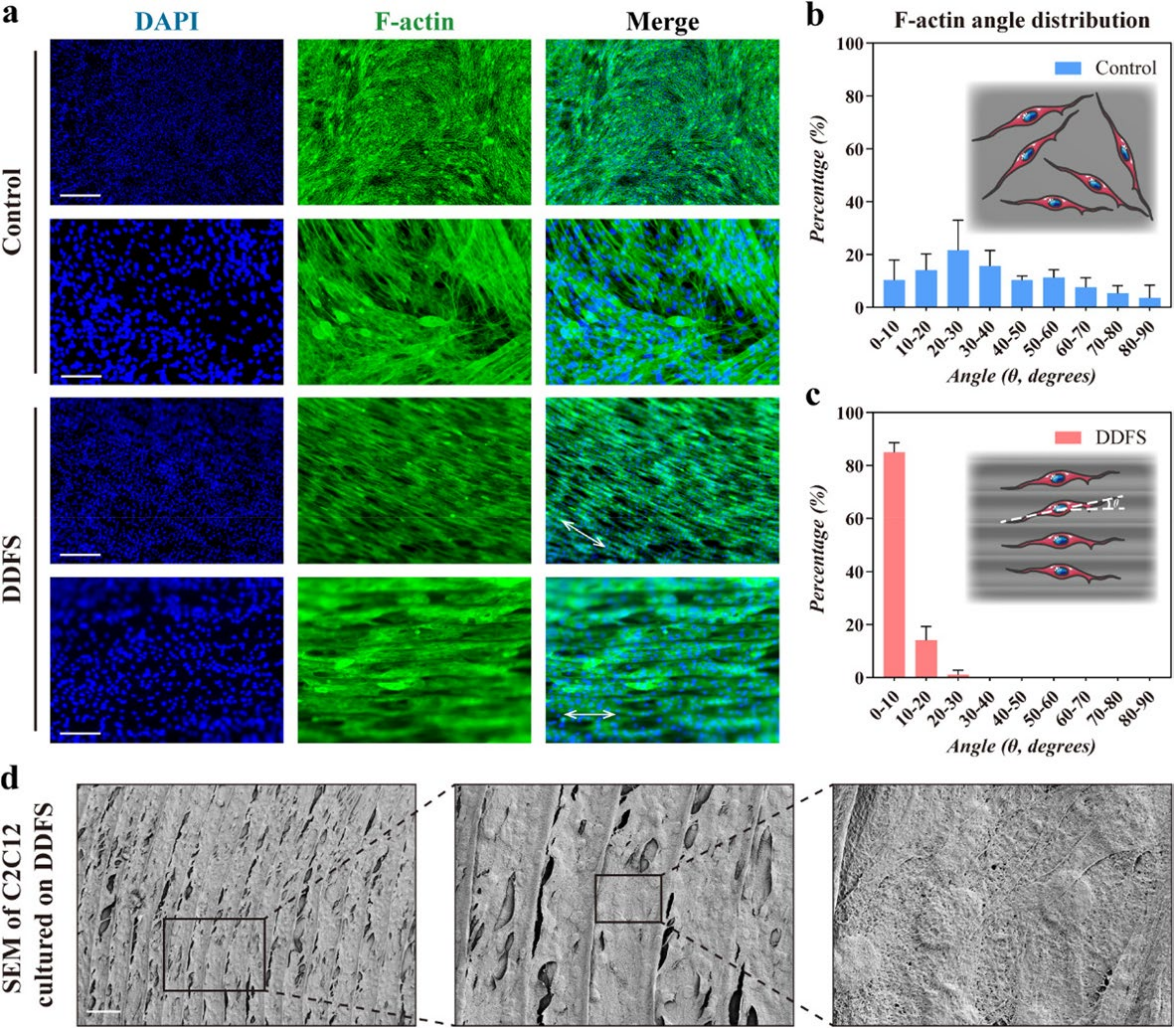
Evaluation of oriented cell growth induction cultured on DDFS. **a** Immunofluorescence staining of C2C12 myotubes cultured on the cell culture plate (control) or DDFSs for filamentous actin (F-actin). White arrows indicated the alignment of myotubes (scale bars: 500 μm and 100 μm, respectively). **b**-**c** The quantitative analysis of orientation angles based on F-actin staining (*n* = 3, mean ± SD). **d** SEM images of C2C12 myotubes cultured on DDFS (scale bar: 100 μm)

The anterior side of the DDFS could be divided into 3 distinguishable patterns (spokes, ridges and spikes) according to different microstructures present on the DDFS [12]. The microstructures of 3 parts of DDFS were demonstrated by SEM, and different microstructures would affect the orientation of cultured cells (Fig. S7). We found that the ridges part of DDFS could induce oriented growth of C2C12 myoblast, which could be observed in the fluorescence images of Calcein AM staining (Fig. S4). To further observe the morphology of cell growth on the DDFS and conduct orientation statistical analysis, immunofluorescence staining for F-actin was performed on the myotubes after 5 days of induction of C2C12 cells with horse serum. It was illustrated that the myotubes elongated along the orientation of the ridges of DDFS, while the myotubes cultured on the plate showed random orientation (Fig. 4a). After the measurement of F-actin-positive cell angle distribution and related statistical analysis, we confirmed that more than 80% of the myotubes cultured on DDFS grew in a uniform direction (Fig. 4b, c). We also performed a SEM test to observe the morphology of the myotubes cultured on DDFS，and found that myotubes mainly grew in an orderly manner along the grooves of DDFS (Fig. 4d).

### Promotion of myogenic differentiation and myotube formation of C2C12 myoblasts by DDFS and SCOS

**Fig. 5:**
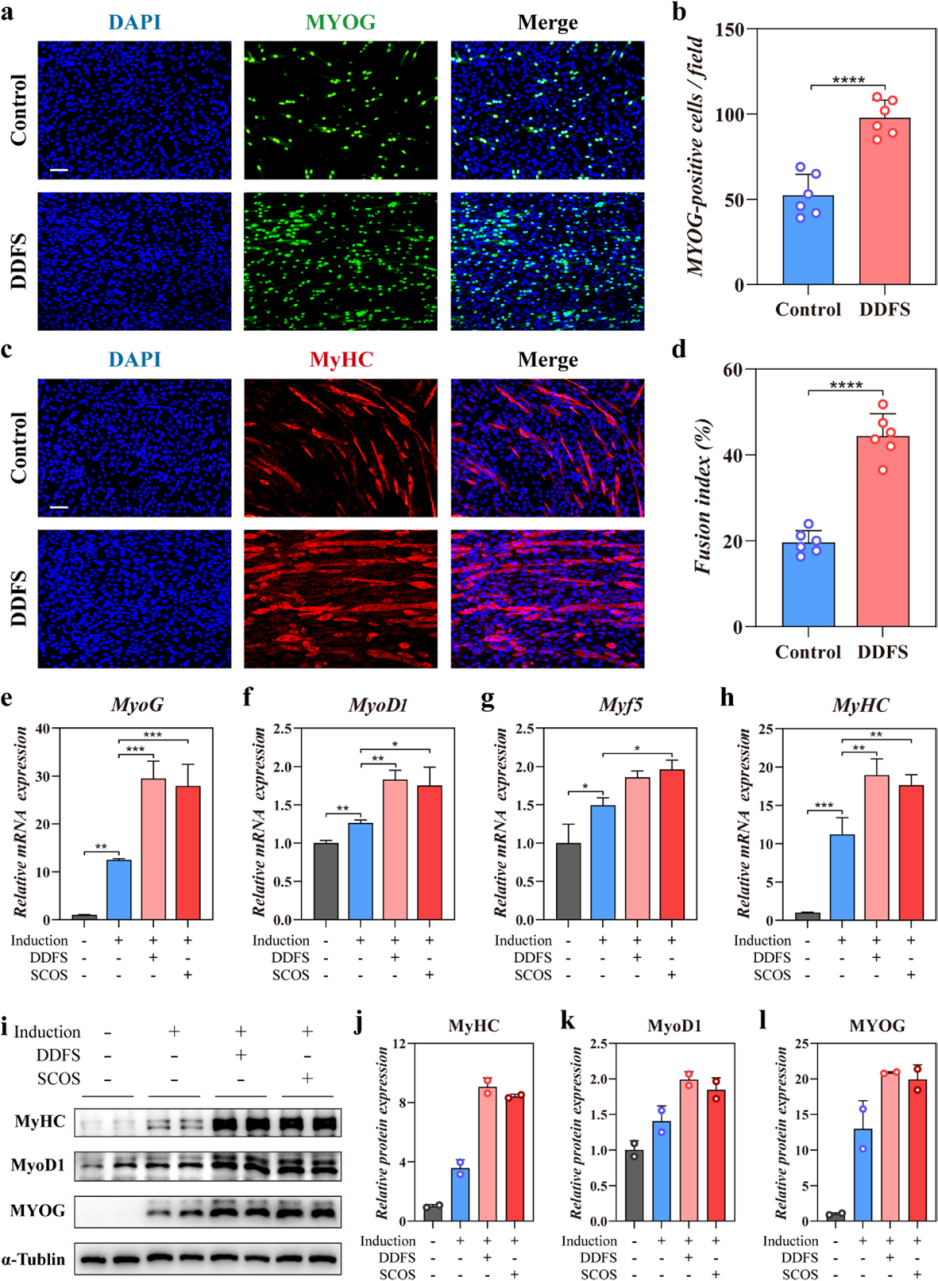
Enhanced myogenic differentiation in C2C12 cultured on DDFS and SCOS. **a** Immunofluorescence staining of C2C12 myotubes cultured on the cell culture plate (control) or DDFSs for myogenic marker myogenin (MYOG). **b** Quantitative analysis of MYOG-positive cells per field (*n* = 6, mean ± SD). **c** Myosin heavy chain (MyHC) immunostaining of C2C12 myotubes cultured on the cell culture plate (control) or DDFSs (scale bar: 100 μm). **d** Fusion index analysis based on MyHC staining (*n* = 6, mean ± SD). **e**-**h** Comparison of mRNA levels of myogenic markers including *MyoG*, *MyoD1*, *Myf5* and *MyHC* by RT-qPCR analysis (*n* = 9, mean ± SD). **i** Western blot assay of protein levels of MyHC, MyoD1 and MyoG. **j**-**l** Quantitative analysis of Western blot results (*n* = 2, mean ± SD). Scale bar: 100 μm. **** *p* < 0.0001, *** *p* < 0.001, ** *p* < 0.01, * *p* < 0.05, by One-way ANOVA or two-tailed Student’s t test

It has been demonstrated that control of cell orientation can upregulate myogenic gene expression and promote formation of myotubes [7, 20, 21]. Thus, we supposed the oriented growth environment provided by the surface of DDFS could promote the differentiation of myoblasts and facilitate the formation of myotubes. Through immunofluorescence staining of myogenin (MYOG), we observed the fusion of MYOG-positive C2C12 after 5 days of induction with horse serum (Fig. 5a). Statistical analysis indicated that a larger number of MYOG-positive cells were found in the DDFS group (Fig. 5b). We calculated the fusion index of C2C12 according to the immunofluorescence staining of Myosin heavy chain (MyHC) and found enhanced fusion index of C2C12 cultured on DDFS (Fig. 5c, d). The MyHC staining also revealed the oriented alignment of myotubes on DDFS. RT-qPCR analysis revealed that C2C12 myoblasts grew on DDFS and SCOS had higher mRNA expression of myogenic markers (Fig. 5e-h). We found that four main myogenic markers expressed by C2C12 in plate, DDFS and SCOS group all increased significantly after induction by horse serum. Differently, the expression level of *MyoG* of C2C12 in the DDFS and SCOS group was more than two-fold higher compared to that in the plate group. Similarly, higher expression levels of myogenic differentiation 1 (*MyoD1*), myogenic factor 5 (*Myf5*), and *MyHC* were also observed in the DDFS and SCOS group. The protein expression of MyHC, MyoD1 and MyoG was further detected through Western blot analysis (Fig. 5i). The expression of these three important myogenic markers in undifferentiated C2C12 was very low. After 5 days of induction, the protein expression of MyHC, MyoD1 and MyoG elevated significantly, and higher expression levels were found in the DDFS and SCOS group (Fig. 5j-l). These results indicated that the DDFS and SCOS could promote the myogenic differentiation of C2C12.

### De novo muscle regeneration and functional restoration induced by SCOS/MT transplants in the VML mouse model

**Fig. 6:**
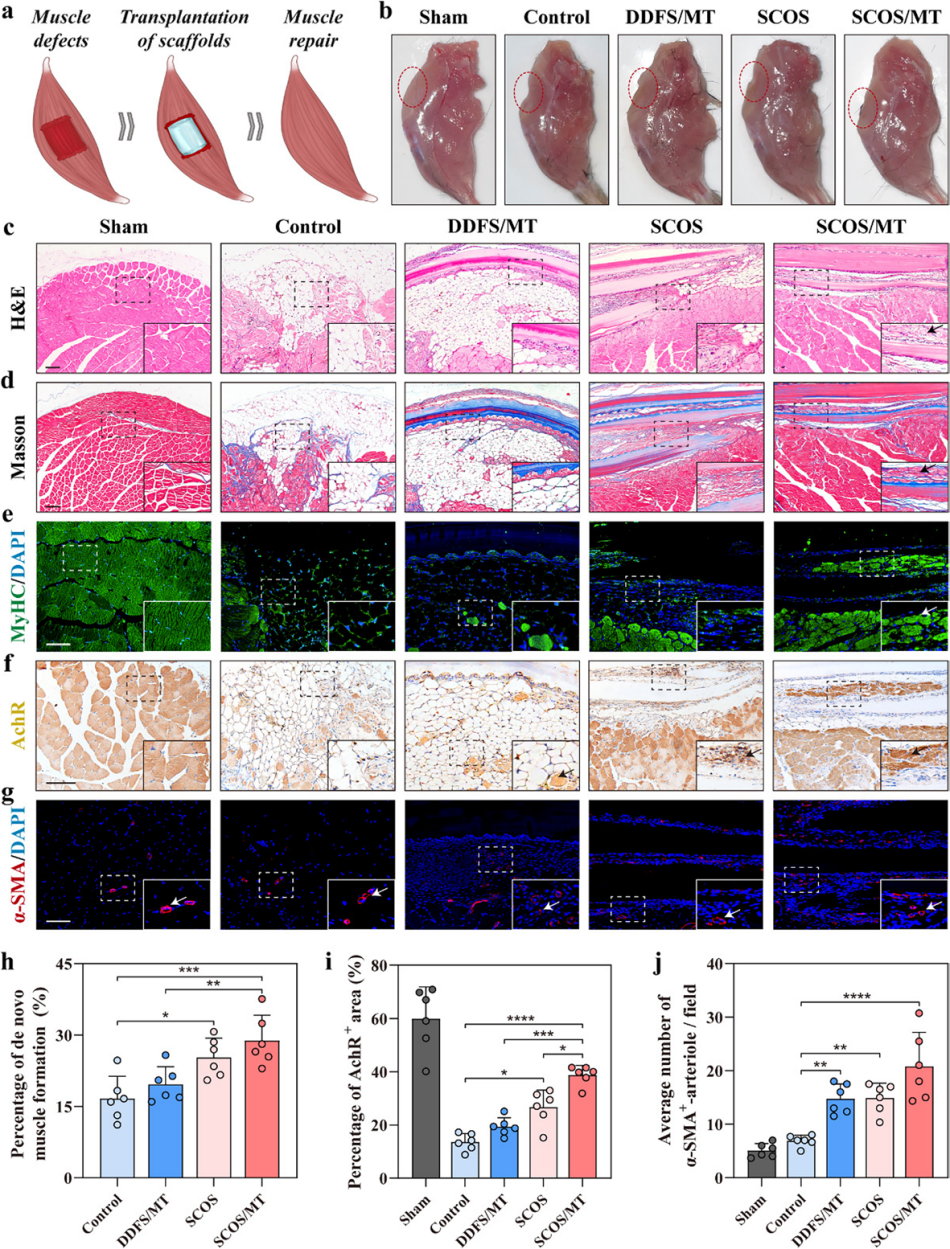
Promotion of muscle regeneration in the mouse model of VML by SCOS/MT constructs transplatation. **a** Schematic illustration of muscle repair assisted by SCOS/MT constructs after VML. **b** Macroscopic view of the transplanted sites treated with control, DDFS/MT, SCOS, and SCOS/MT after 8 weeks. **c**-**d** Representative H&E and Masson trichrome stained images of muscle tissues with sham or VML injury in quadriceps femoris muscles 8 weeks following transplantation. **e** Immunofluorescence staining of MyHC. **f** Immunohistochemical staining of AchR. **g** Immunofluorescence staining of α-SMA. **h** The quantitative results of de novo muscle formation according to MyHC staining. **i**-**j** The quantitative results of AchR-positive area and α-SMA-positive vessels, respectively (*n* = 5–6, mean ± SD). Scale bar: 100 μm. **** *p* < 0.0001, *** *p* < 0.001, ** *p* < 0.01, * *p* < 0.05, by One-way ANOVA test

For in vivo experiment, we first added C2C12 cell suspension on the SCOSs. After cell adherence, the SCOSs were immersed into the culture medium for self-curling. After induction of myogenic differentiation for 5 days, C2C12 myoblasts cultured on the SCOSs differentiated into myotubes, and the SCOS/MT transplants for VML treatment were prepared. In the meantime, DDFSs loaded with myotubes (DDFS/MT) were also generated. The mouse VML injury model was constructed through excising quadriceps femoris muscles by surgical resection to 75% muscle loss that could not recover without any treatment (Fig. 6a). All mice were divided into 5 groups, including mice without VML injury (sham), VML mice without treatment (control) or treated with DDFS/MT, SCOS or SCOS/MT. The transplants were placed on the ablated region and sutured under the fascia. To test the survival of C2C12 after transplantation, we employed RFP-C2C12. SCOSs loaded with RFP-C2C12 were implanted in VML mice, and C2C12 showed good survival ability 7 days after implantation according to fluorescence detection (Fig. S8).

**Fig. 7:**
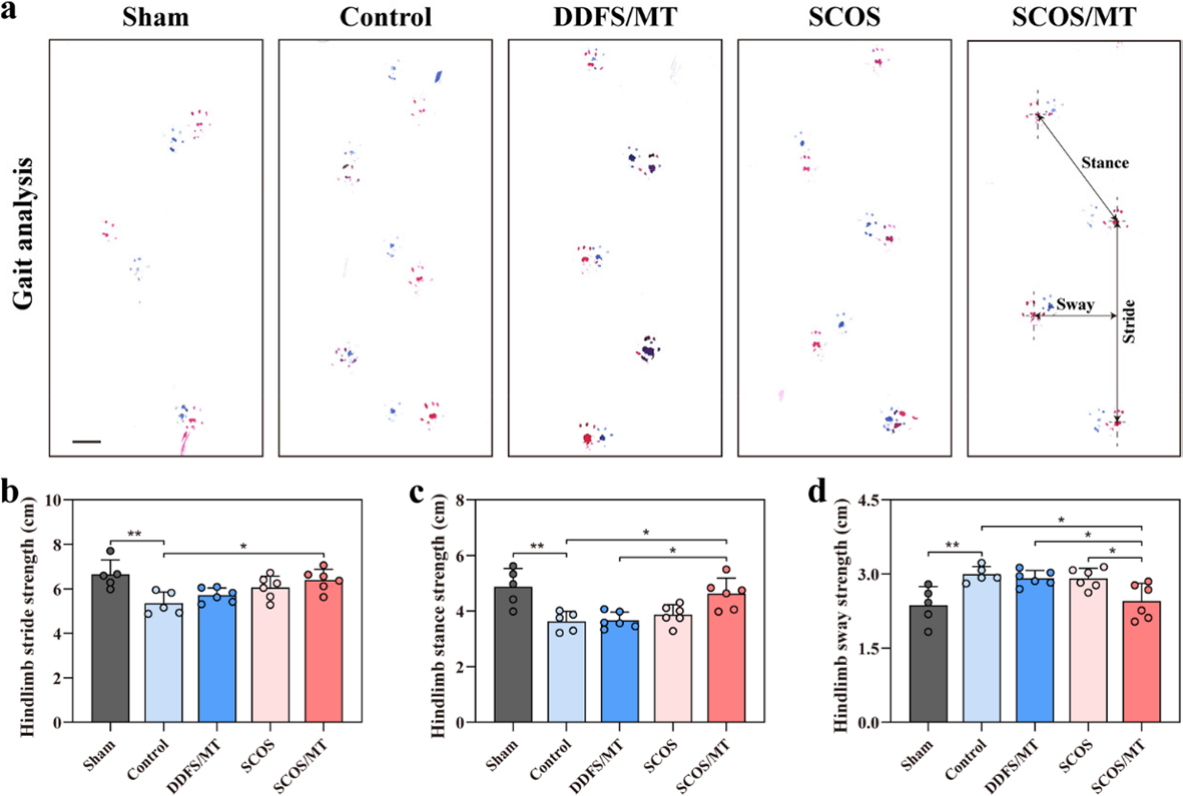
Functional improvement of injured muscle in the mouse model of VML by SCOS/MT constructs transplatation. **a** Representative footsteps of gait analysis at week 8 (scale bar: 1 cm). **b**-**d** Quantitative analysis of impaired hindlimb stride length, hindlimb stance length, and hindlimb sway length (*n* = 5–6, mean ± SD). ** *p* < 0.01, * *p* < 0.05, by One-way ANOVA test

Eight weeks after the operation, there were obvious defects in quadriceps femoris muscles of VML mice in the control group according to the macroscopic view, while defects were filled by transplants in DDFS/MT, SCOS and SCOS/MT groups (Fig. 6b). To better evaluate the therapeutic effects of the SCOS/MT, H&E and Masson trichrome staining were conducted. We found that all transplants were kept in place and SCOS could maintain the curling shape (Fig. 6c). Results showed that significant fat deposition was observed in control and DDFS/MT groups, and few newly formed muscular tissue was found in control and SCOS groups (Fig. 6c, d). In contrast, de novo muscular tissue formation was induced in the SCOS/MT group. Masson trichrome staining indicated fibrous tissue formation in the ablated region of all VML mice, and reduced fibrosis could be found in the SCOS/MT group (Fig. 6c). Immunofluorescence staining of MyHC revealed formation of MyHC-positive myofibers, and the quantification result showed significantly more de novo muscle formation in the SCOS/MT group than other groups (Fig. 6e, h). In addition, immunostaining revealed that SCOS/MT constructs induced higher expression of acetylcholine receptor (AchR) compared to that of other groups, indicating more innervation in the regenerated muscle tissues (Fig. 6f, i). Compared with the sham group, all VML groups had more alpha-smooth muscle actin (α-SMA)-positive vessels, while the SCOS/MT group generated more α-SMA-positive vessels than other groups, indicating improved vascularization (Fig. 6g, j). Then, we assessed the functional performance of impaired hindlimbs of mice with VML via footprint gait analysis after 8 weeks (Fig. 7a-d). The mice with the SCOS/MT transplants showed increased stance and stride lengths compared with the control group (Fig. 7b, c). In the meantime, the SCOS/MT group exhibited a significantly lower sway length than control mice, indicating an improvement in their walking behaviors. Besides, H&E staining and serum biochemical analysis revealed that the transplants had no toxicity (Fig. S9a, b). These above results demonstrated that SCOS/MT constructs could promote muscle regeneration and functional recovery in the VML mouse model.

## Discussion

To overcome the shortcomings of current treatment for volumetric muscle loss, development of tissue engineering scaffolds that can induce muscle regeneration is a promising strategy. Due to the highly ordered alignment of cellular and extracellular structure, it is critical to mimic the 3D oriented growth of muscle tissues. Here, inspired by the microstructure of fish scales, we developed a self-curling 3D oriented scaffold composed of DDFS and fish GelMA for VML treatment. In fact, fish scales have been proved to have great biocompatibility and low immunogenicity, and have been employed to exploit tissue engineering scaffolds for cornea and skin reconstitution [10, 12]. Furthermore, we found that the microgroove structure on the surface of fish scales could induce excellent oriented cell growth (Fig. 4). In fact, various biomaterials with microgroove structure have been developed to induce cell orientation [22–25]. Whereas, the planar structure of fish scales makes it difficult to apply fish scales alone to develop scaffolds for 3D tissue repair. In the present study, by combining fish scales and fish GelMA, we fabricated a hybrid scaffold that could self-curl into a 3D structure due to different swelling properties of two compositions. Our data revealed that this SCOS could promote myogenic differentiation of C2C12 myoblasts and SCOS loaded with myotube could induce muscle regeneration and functional restoration in vivo.

According to histological analysis and DNA content detection, fish scales were successfully decellularization after 0.1% SDS treatment (Fig. 2a and S1a). Decellularized fish scales are mainly consisted of hydroxyapatite and type I collagen [16]. As an extracellular matrix component, type I collagen is ubiquitously distributed in various tissues including skeletal muscles, and has been proved to stimulate myogenesis [26]. Thus, type I collagen of fish scales, rather than hydroxyapatite, is suitable for skeletal muscle engineering. The collagen quantification assay revealed that collagen component of fish scales could be retained after decalcification (Fig. S1b). Mechanical test revealed that Young’s modulus of DDFS significantly decreased, which meant DDFSs became softer and more similar to the stiffness of mouse muscle. Furthermore, the ridges part of DDFS still owned the original microgroove structures that could induce oriented cell growth (Fig. 4 and S7). It has been reported that fish collagen derived gelatin can be applied in the generation of GelMA. According to previous studies, fish GelMA hydrogels have a higher swelling ratio compared with conventional porcine skin GelMA hydrogels on account of the fewer content of hydrophobic amino acids in fish gelatin, which means fish GelMA hydrogels can provide more curling power when combining with DDFSs [27, 28] Fish GelMA hydrogels with concentrations from 2.5 to 15% all showed higher mass swelling ratios than DDFSs (Fig. 2g). Although the swelling properties of fish GelMA hydrogels decreased with the increasing concentration, we found SCOS composed of 5% fish GelMA could curl better, which may be attributed to poor mechanical properties of hydrogels with 2.5% fish GelMA (Fig. S3). In addition, DDFS and SCOS both exhibited good biocompatibility and degradability, which was critical to tissue engineering (Fig. 3 and S5).

The regulation of cells alignment is of great significance for functional regeneration and maturation of oriented tissues like skeletal muscle tissue [14, 29–32]. Due to the oriented growth induced by DDFS, SCOS was demonstrated to promote myogenic differentiation of C2C12 myoblasts in vitro. Compared with C2C12 cultured on plates, C2C12 cultured on SCOSs had higher mRNA and protein levels of myogenic markers (Fig. 5). Furthermore, SCOS loaded with C2C12 induced myotubes exhibited great potential to promote muscle regeneration in vivo. Although skeletal muscle can spontaneously regenerate, recovery from severe injuries like VML is hard to achieve. As shown in H&E staining, the defects of muscle tissues were filled with adipose and connective tissues in the control group, while de novo muscle formation could be found in the SCOS/MT group. In addition, immunostaining of AchR revealed that transplantation of SCOS/MT also promote innervation in new formed muscle tissues, and innervation is critical for functional recovery of injured muscle tissues. Since skeletal muscle is highly metabolic, the muscle tissue healing process also needs revascularization. More α-SMA-positive vessels in the SCOS/MT group showed that microvessels could grow into the scaffolds and peripheral tissues, which meant nutrients necessary for tissue regeneration were accessible (Fig. 6). Functional restoration is critical for muscle regeneration. The SCOS/MT group performed better in gait analysis, revealing satisfying muscle function (Fig. 7).

The present study proposed a convenient approach to fabricate tissue engineering scaffolds for skeletal muscle regeneration. However, several limitations of this study should also be pointed out. First of all, although it has been reported that C2C12 myoblasts can be applied in muscle tissue engineering, more suitable seed cells should be developed for further research [33–36]. In recent years, induced myogenic progenitor cells (iMPCs) have been generated, which may be better for muscle regeneration [37]. Secondly, our research did not focus on the cell signaling pathways that contributed to muscle regeneration during transplantation. Fish scale derived scaffolds have been proved to adjust macrophage response, and this process may also contribute to muscle regeneration [10]. Thirdly, some bioactive factors can be cooperated with our scaffolds. Suitable bioactive factors can be loaded by GelMA, and may further promote muscle generation in vivo. Fourthly, more time points of in vivo experiments should be added in further research to observe the regeneration process.

## Conclusions

In this study, we developed a novel SCOS composed of fish derived GelMA and fish scales for skeletal muscle regeneration. Soft DDFSs with oriented microgroove structures were first fabricated by decellularization and decalcification, which could induce oriented growth of C2C12 myoblasts. Besides, fish GelMA hydrogels with different swelling properties were integrated on the DDFSs via in-situ UV irradiation to make the scaffolds own self-curling function and thus achieve a 3D structure. Additionally, fish derived biomaterials had low immunogenicity and great biocompatibility, making them suitable for tissue engineering. It was demonstrated through morphology analysis and detection of myogenic markers that the organized cell alignment contributed to the myogenic differentiation and promoted the formation of myotubes. In vivo experiments further revealed that SCOSs loaded with myotubes differentiated from C2C12 cells could promote muscle regeneration in mice with skeletal muscle injuries. With all these features, such SCOSs presented in our study are anticipated to provide a new idea for the application of fish derived biomaterials and realize clinical translation after future pre-clinical studies.

## Supplementary Information

**Additional file 1: Table S1.** Primers used for qPCR. **Fig. S1.** DNA (a) and collagen (b) content of fish scales before and after decellularization and decalcification (*n* = 3, mean ± SD). c) DAPI staining of fish scales before and after decellularization (scale bar: 100 μm). d) EDS of results of fish scales after decellularization and decalcification. The yellow boxes indicate peaks of calcium (Ca) and phosphorus (P) elements. e) The degradation process of fish scales after decellularization and decalcification (*n* = 3, mean ± SD). **Fig. S2.** Representative ^1^H NMR spectrum of fish gelatin and GelMA. Peaks indicate methacrylamide grafts of lysine (a, b) and unreacted lysine groups (c). **Fig. S3.** a) Representative images of SCOSs composed of different concentrations of fish GelMA (scale bar: 2 mm). b Quantification of shape change degrees of SCOSs (*n* = 3, mean ± SD). c The mechanical test of SCOSs (*n* = 3, mean ± SD). **Fig. S4.** The in vitro biocompatibility of DDFS. a) Live/Dead cellular staining of induced C2C12 myotubes cultured on the cell culture plate (control) or DDFSs (scale bar: 500 μm). b) Quantitative results of dead cells (*n* = 5, mean ± SD). **Fig. S5.** The in vivo biocompatibility of DDFS and SCOS. a-b) H&E and Masson trichrome staining of skin tissues from control mice or mice implanted with DDFS or SCOS for 2 weeks (scale bar: 100 μm). c) Immunohistochemical staining of TNF-α. d) Biochemical detection of serum from control mice or mice implanted with DDFS or SCOS for 2 weeks (*n* = 3, mean ± SD). **Fig. S6.** General observation of SCOS before (a) and after (b) implantation for 4 weeks (scale bar: 5 mm). **Fig. S7.** SEM images of different regions of DDFS (scale bar: 100 μm) and Calcein AM staining of C2C12 myoblasts cultured on different regions of DDFS (scale bar: 500 μm). White arrows indicated the alignment of C2C12 myoblasts. **Fig. S8.** Fluorescence signals detection after the implantation of SCOS loaded with RFP-C2C12 for 7 days. **Fig. S9.** H&E images of main organs (a) and serum biochemical detection (b) from VML mice in different groups. Scale bar: 100 μm.

## Data Availability

The data that support the findings of this study are available from the corresponding author upon reasonable request.

## Abbreviations

VML: Volumetric muscle loss
3D: Three-dimensional
SCOS: Self-curling 3D oriented scaffold
GelMA: Gelatin methacrylate
DDFS: Decellularized and decalcified fish scales
SCOS/MT: SCOSs loaded with induced myotubes
DDFS/MT: DDFSs loaded with induced myotubes
SEM: Scanning electron microscope
EDS: Energy dispersive spectrometer
FTIR: Fourier-transformed infrared
CCK-8: Cell Counting Kit-8
PI: Propidium iodide
SBF: Simulated body fluid
H&E: Hematoxylin & eosin
MYOG: Myogenin
MyHC: Myosin heavy chain
α-SMA: α-smooth muscle actin
AchR: Acetylcholine receptor
MyoD1: Myoblast determination protein 1
RT-qPCR: Real-time quantitative polymerase chain reaction
