# Supplementary material for: Self-curling 3D oriented scaffolds from fish scales for skeletal muscle regeneration
Source: Biomater Res. 2022 Dec 22;26:87. doi: 10.1186/s40824-022-00335-w (PMC9773491; doi:10.1186/s40824-022-00335-w)
Supplement: Supplementary file 1 — Additional file 1: Table S1. Primers used for qPCR. Fig. S1. DNA (a) and collagen (b) content of fish scales before and after decellularization and decalcification (n = 3, mean ± SD). c) DAPI staining of fish scales before and after decellularization (scale bar: 100 μm). d) EDS of results of fish scales after decellularization and decalcification. The yellow boxes indicate peaks of calcium (Ca) and phosphorus (P) elements. e) The degradation process of fish scales after decellularization and decalcification (n = 3, mean ± SD). Fig. S2. Representative 1H NMR spectrum of fish gelatin and GelMA. Peaks indicate methacrylamide grafts of lysine (a, b) and unreacted lysine groups (c). Fig. S3. a) Representative images of SCOSs composed of different concentrations of fish GelMA (scale bar: 2 mm). b Quantification of shape change degrees of SCOSs (n = 3, mean ± SD). c The mechanical test of SCOSs (n = 3, mean ± SD). Fig. S4. The in vitro biocompatibility of DDFS. a) Live/Dead cellular staining of induced C2C12 myotubes cultured on the cell culture plate (control) or DDFSs (scale bar: 500 μm). b) Quantitative results of dead cells (n = 5, mean ± SD). Fig. S5. The in vivo biocompatibility of DDFS and SCOS. a-b) H&E and Masson trichrome staining of skin tissues from control mice or mice implanted with DDFS or SCOS for 2 weeks (scale bar: 100 μm). c) Immunohistochemical staining of TNF-α. d) Biochemical detection of serum from control mice or mice implanted with DDFS or SCOS for 2 weeks (n = 3, mean ± SD). Fig. S6. General observation of SCOS before (a) and after (b) implantation for 4 weeks (scale bar: 5 mm). Fig. S7. SEM images of different regions of DDFS (scale bar: 100 μm) and Calcein AM staining of C2C12 myoblasts cultured on different regions of DDFS (scale bar: 500 μm). White arrows indicated the alignment of C2C12 myoblasts. Fig. S8. Fluorescence signals detection after the implantation of SCOS loaded with RFP-C2C12 for 7 days. Fig. S9. H&E images of main organs (a) and [file 40824_2022_335_MOESM1_ESM.docx]

**Self-curling** **3D oriented scaffolds** **from** **fish scales for skeletal muscle regeneration**

Yong Shi ^1,4^, Xiaoxuan Zhang ^2^, Rui Liu ^3^, Xiaoyan Shao ^1^, Yuanjin Zhao ^1,2,3^*, Zhuxiao Gu ^1,3,4^*, Qing Jiang ^1,4^*

1. State Key Laboratory of Pharmaceutical Biotechnology, Division of Sports Medicine and Adult Reconstructive Surgery, Department of Orthopedic Surgery, Nanjing Drum Tower Hospital, The Affiliated Hospital of Nanjing University Medical School, 321 Zhongshan Road, Nanjing 210008, Jiangsu, PR China
2. State Key Laboratory of Bioelectronics, School of Biological Science and Medical Engineering, Southeast University, Nanjing 210096, China
3. Department of Rheumatology and Immunology, Institute of Translational Medicine, The Affiliated Drum Tower Hospital of Nanjing University Medical School, Nanjing, 210002, China.
4. Branch of National Clinical Research Center for Orthopedics, Sports Medicine and Rehabilitation, PR China

*Correspondence: [yjzhao@seu.edu.cn](mailto:yjzhao@seu.edu.cn); [gzx@seu.edu.cn](mailto:gzx@seu.edu.cn); [qingj@nju.edu.cn](mailto:qingj@nju.edu.cn)

**Table S1. Primers used for qPCR**

| Target gene | Primer sequence (5’ to 3’)  Forward Reverse | |
| --- | --- | --- |
| Mouse MyoG | CAGTGAATGCAACTCCCACAG | TGGACGTAAGGGAGTGCAGA |
| Mouse MyoD1 | GACGGCTCTCTCTGCTCC | AAGTGTGCGTGCTCCTCC |
| Mouse Myf5 | CGGATCACGTCTACAGAGCC | GCAGGAGTGATCATCGGGAG |
| Mouse MyHC | GAGTTCATTGACTTCGGGATGG | TGCTGCTCATACAGCTTGTTCTTG |


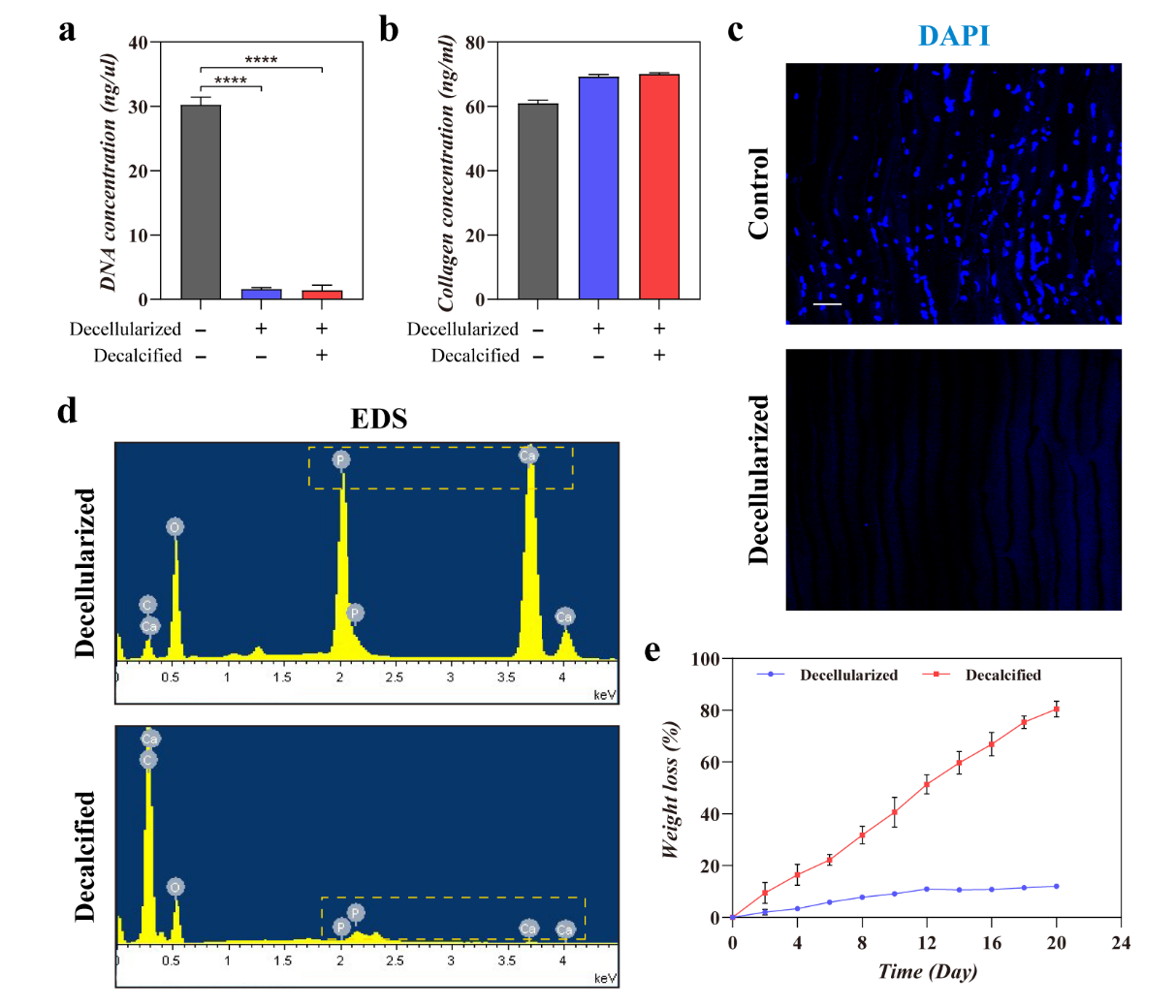


**Figure S1.** DNA (a) and collagen (b) content of fish scales before and after decellularization and decalcification (n = 3, mean ± SD). c) DAPI staining of fish scales before and after decellularization (scale bar: 100 μm). d) EDS of results of fish scales after decellularization and decalcification. The yellow boxes indicate peaks of calcium (Ca) and phosphorus (P) elements. e) The degradation process of fish scales after decellularization and decalcification (n = 3, mean ± SD).

**
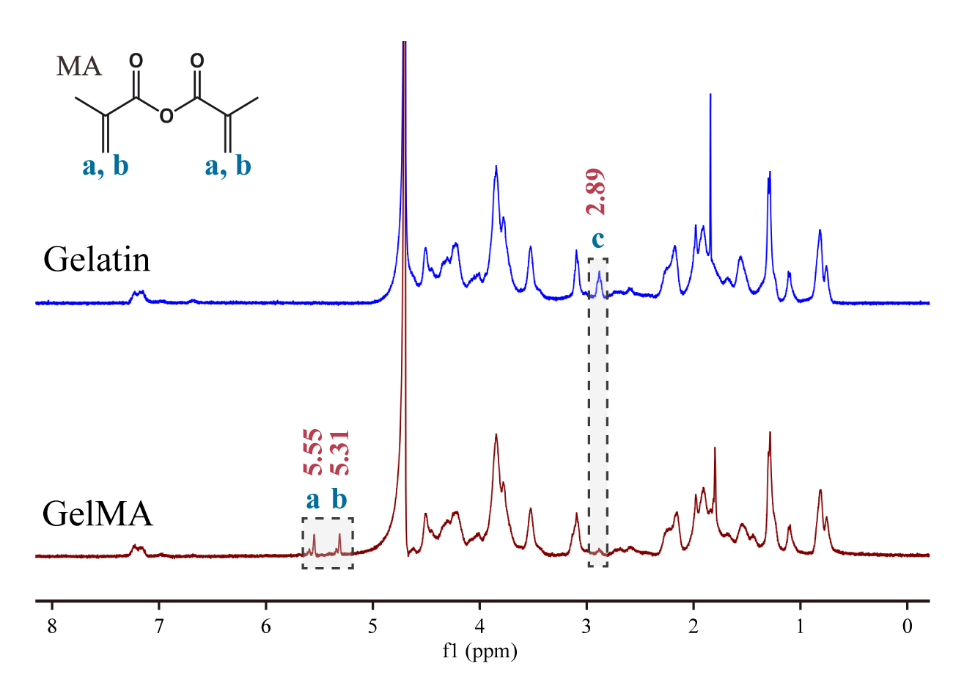
**

**Figure S2.** Representative ^1^H NMR spectrum of fish gelatin and GelMA. Peaks indicate methacrylamide grafts of lysine (a, b) and unreacted lysine groups (c).


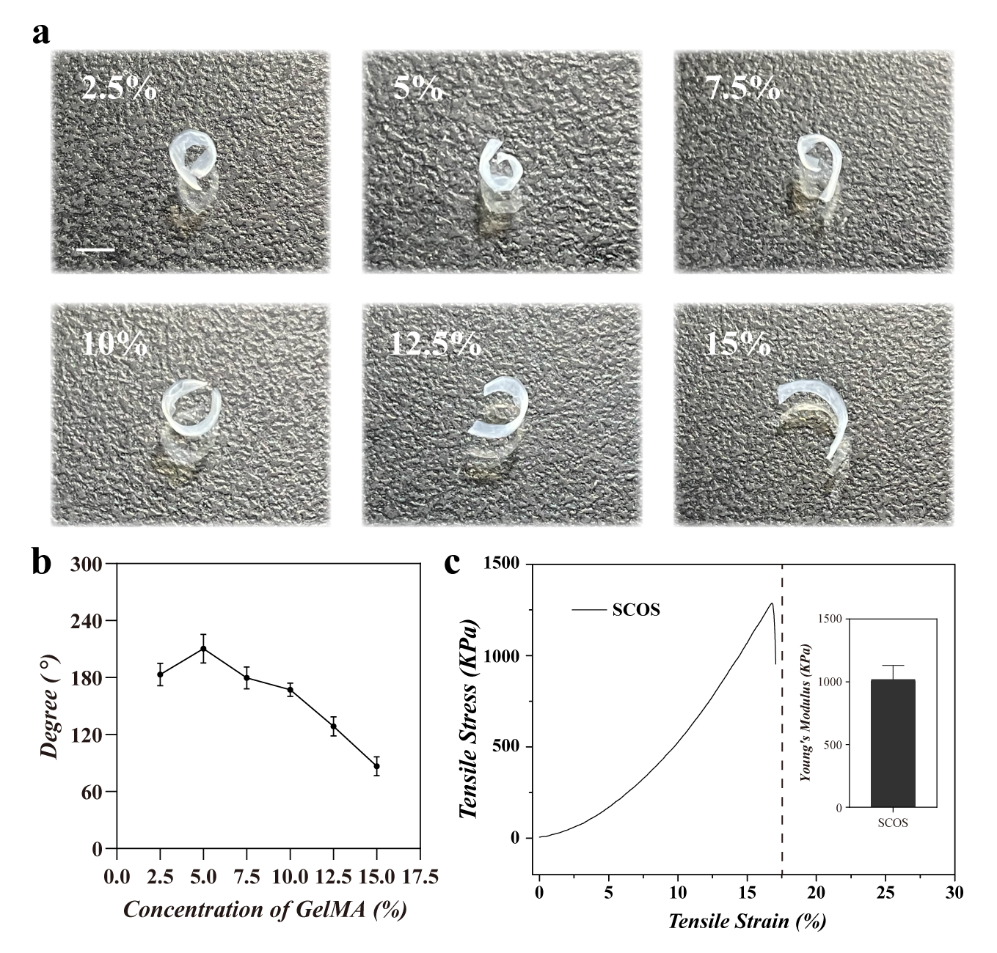


**Figure S3.** a) Representative images of SCOSs composed of different concentrations of fish GelMA (scale bar: 2 mm). b) Quantification of shape change degrees of SCOSs (n = 3, mean ± SD). c) The mechanical test of SCOSs (n = 3, mean ± SD).


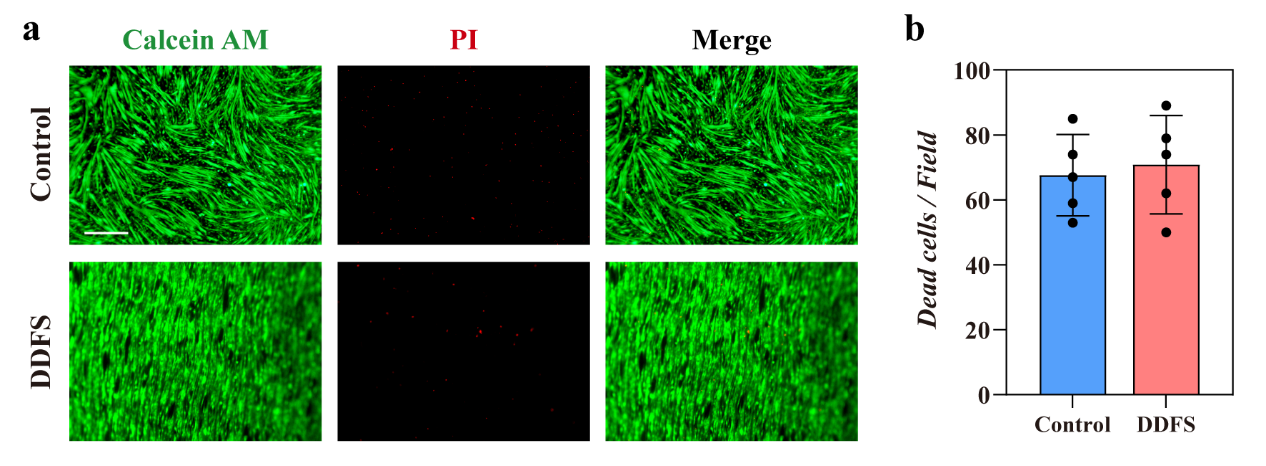


**Figure S4.** The in vitro biocompatibility of DDFS. a) Live/Dead cellular staining of induced C2C12 myotubes cultured on the cell culture plate (control) or DDFSs (scale bar: 500 μm). b) Quantitative results of dead cells (n = 5, mean ± SD).


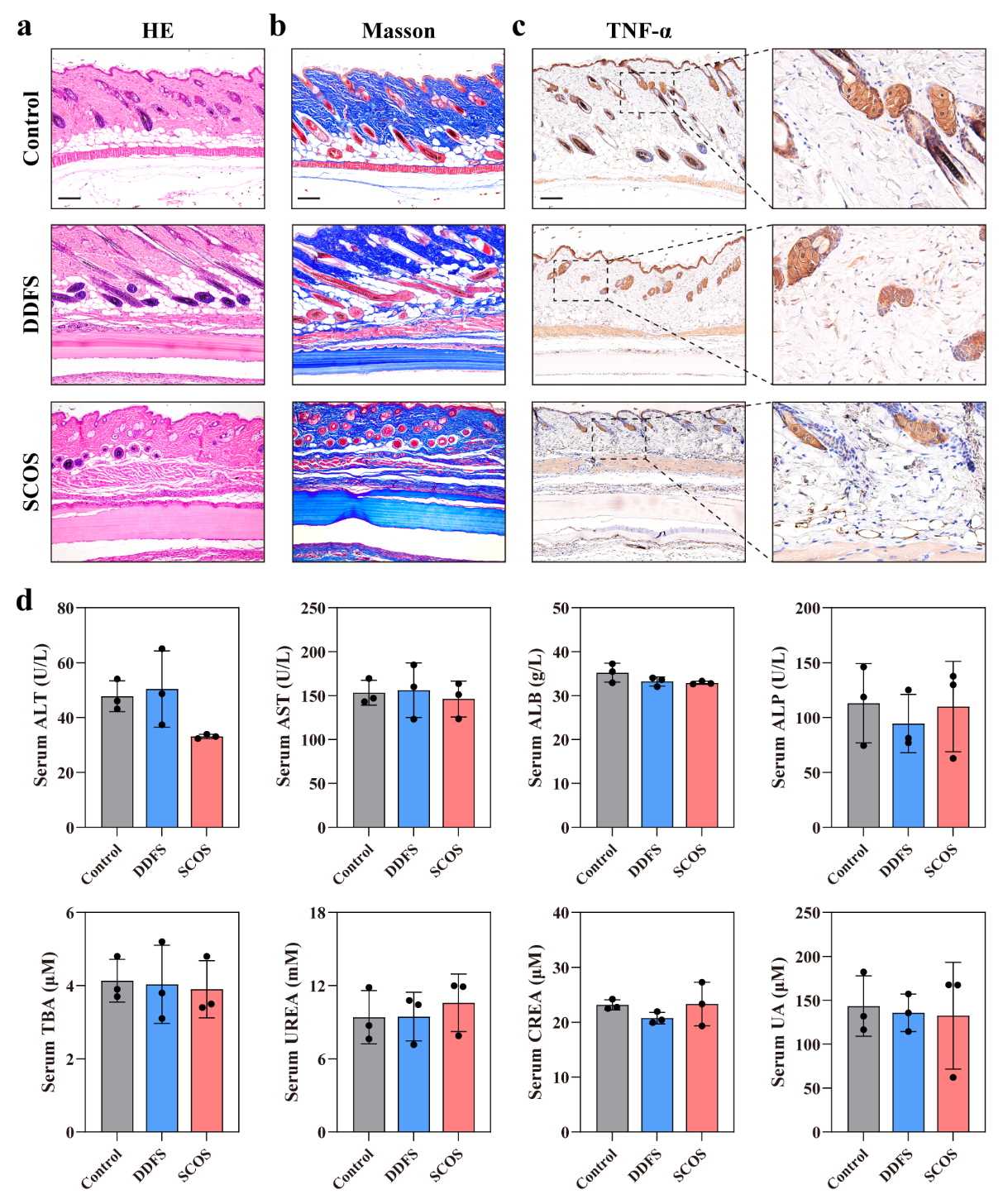


**Figure S5.** The in vivo biocompatibility of DDFS and SCOS. a-b) H&E and Masson trichrome staining of skin tissues from control mice or mice implanted with DDFS or SCOS for 2 weeks (scale bar: 100 μm). c) Immunohistochemical staining of TNF-α. d) Biochemical detection of serum from control mice or mice implanted with DDFS or SCOS for 2 weeks (n = 3, mean ± SD).


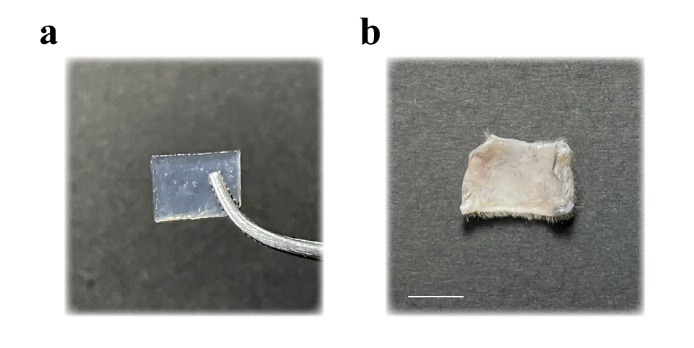


**Figure S6.** General observation of SCOS before (a) and after (b) implantation for 4 weeks (scale bar: 5 mm).


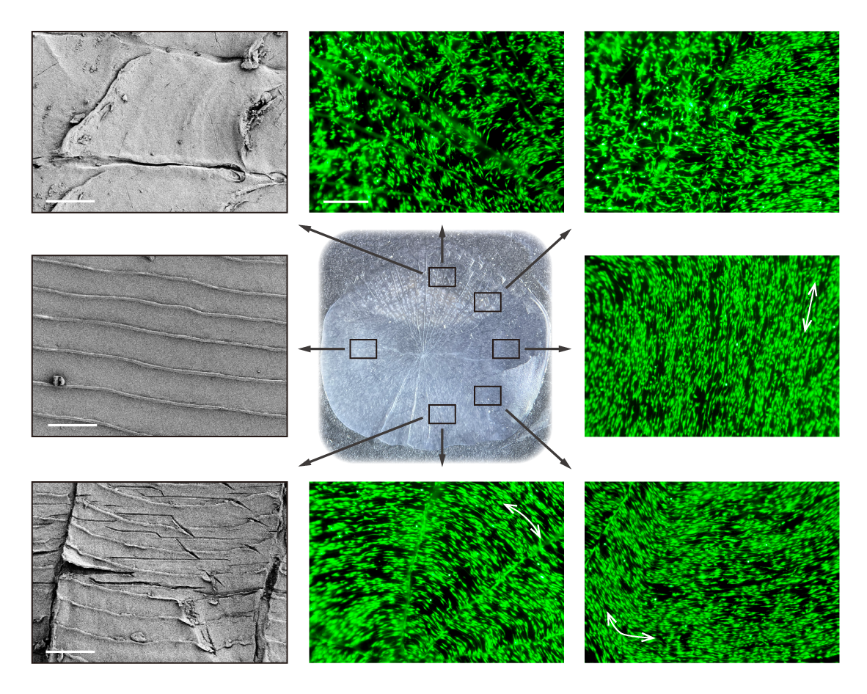


**Figure S7.** SEM images of different regions of DDFS (scale bar: 100 μm) and Calcein AM staining of C2C12 myoblasts cultured on different regions of DDFS (scale bar: 500 μm). White arrows indicated the alignment of C2C12 myoblasts.


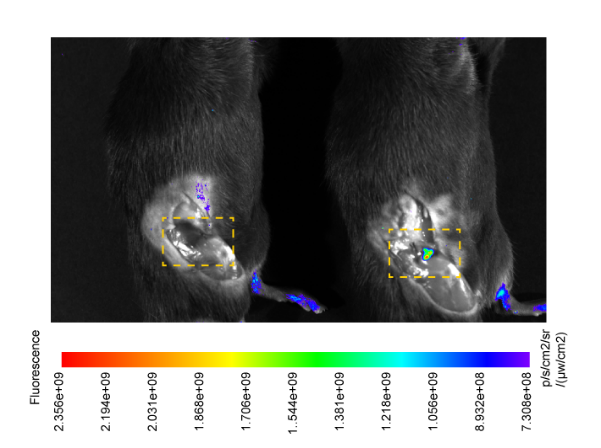


**Figure S8.** Fluorescence signals detection after the implantation of SCOS loaded with RFP-C2C12 for 7 days.


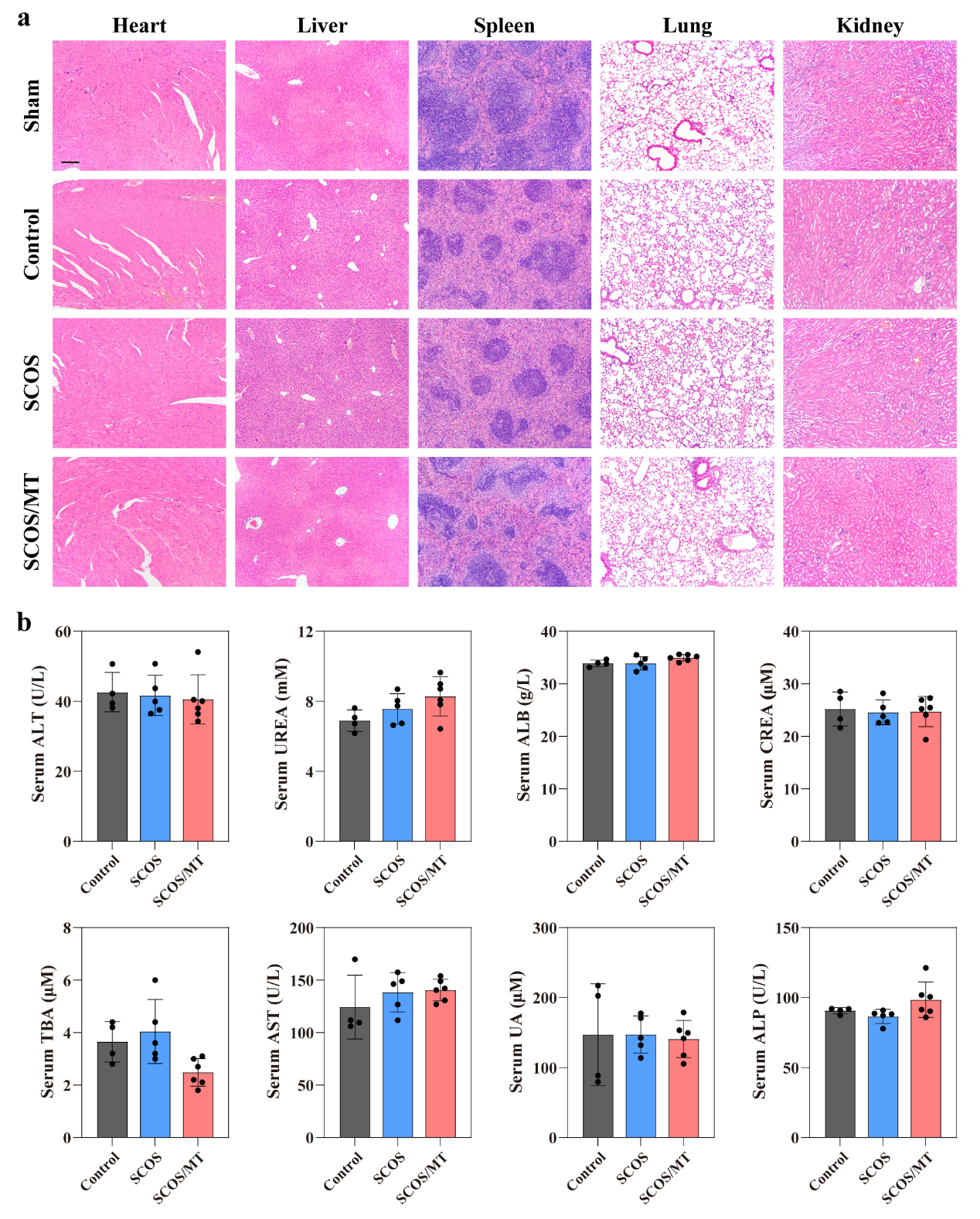


**Figure S9.** H&E images of main organs (a) and serum biochemical detection (b) from VML mice in different groups. Scale bar: 100 μm.
